# Osteoporosis, diabetes, and hypertension are major risk factors for mortality in older adults: an intermediate report on a prospective survey of 1467 community-dwelling elderly healthy pensioners in Switzerland

**DOI:** 10.1186/s12877-018-0809-0

**Published:** 2018-05-15

**Authors:** Jean-Pierre Gutzwiller, Jean-Pierre Richterich, Zeno Stanga, Urs E. Nydegger, Lorenz Risch, Martin Risch

**Affiliations:** 1 Magendarm Thalwil AG, Zürcherstrasse 61, CH-8800 Thalwil, Switzerland; 2 Department of Diabetes, Endocrinology, Nutritional Medicine and Metabolism, Bern University Hospital and University of Bern, Bern, Switzerland; 3 Divisions of Clinical Chemistry and Haematology, Labormedizinisches Zentrum Dr. Risch, Waldeggstrasse, Liebefeld b, Bern, Switzerland; 4 Central Laboratory, Kantonsspital Graubünden, Loëstrasse, 170 Chur, Switzerland; 5 University of Basel, Klingelbergstrasse 61, 4056 Basel, Switzerland; 6 Department of General Internal Medicine, Bern University Hospital and University of Bern, Bern, Switzerland; 7 Labormedizinisches Zentrum Dr. Risch, Landstrasse, Schaan, Liechtenstein

**Keywords:** Osteoporosis, Mortality, Diabetes, Hypertension, Pensioners

## Abstract

**Background:**

Osteoporosis is an important morbidity factor for ageing populations in developed countries. However, compared to the amount of information available on diabetes and cardiovascular disease, little is known about the direct impact of osteoporosis on general mortality in older age.

**Methods:**

We obtained data from a prospective population-based cohort of pensioners from the SENIORLAB study who were subjectively healthy. The inclusion criteria were an age of at least 60 years and Swiss residence. We assessed and analysed clinical measures, voluntary reports, and laboratory values.

**Results:**

In total, 1467 subjects were included in the cohort. The mean follow-up time was 3.68 years (95% confidence interval, 3.64–3.71). The ages of the included participants ranged from 60 to 99 years. At follow-up, there were 1401 survivors, and 66 participants had died. According to the multivariate analysis (Cox regression), osteoporosis was the most important risk factor for all-cause mortality (hazard ratio, 4.46; 95% confidence interval, 1.82–10.91), followed by diabetes (hazard ratio, 2.17; 95% confidence interval, 1.04–4.52) and hypertension (hazard ratio, 1.81; 95% confidence interval, 1.09–3.03).

**Conclusions:**

Osteoporosis is a major risk factor for all-cause mortality in a subjectively healthy senior population, followed by type 2 diabetes mellitus and hypertension. Osteoporosis should be more actively diagnosed in healthy pensioners before they develop osteoporosis-associated health incidents.

**Trial registration:**

The present study was registered in the International Standard Randomized Controlled Trial Number registry: ISRCTN53778569.

## Background

In developed countries, the number of seniors over 60 years of age has grown over the last century. In Switzerland, the proportion of adolescents in relation to the total population fell from 40.7% in 1900 to 20.2% in 2014 and was accompanied by a rise in the proportion of pensioners (aged over 64 years) from 5.8% (1900) to 17.8% (2014). This rate is expected to increase to 26% by 2045 [1]. In addition, the generation over 64 years of age has become wealthier in recent decades, and an estimated 80% of homeowners are above 65 years of age [2].

The aging generation is in better health than in the past. The Swiss Federal Statistical Office maintains mortality statistics; these data include the prevalence of multimorbid terminal phases, among which one nosological entity is assigned as the cause of death [1]. Currently, little is known about the risk factors for mortality in the older generation. The best evidence is from the Cardiovascular Health Study, which was published in 1998 [3]. That study demonstrated that arteriosclerosis (i.e., aortic stenosis and stenosis of the internal carotid artery) was a principal risk factor for mortality, accounting for a two- to fivefold excess mortality risk. The report found that systolic hypertension (over 169 mmHg, a 2.4-fold excess mortality risk), use of diuretics, renal failure, and diabetes with fasting glucose > 7.2 mmol/L (approximately twofold) were less important factors. That study also identified both age and smoking as minor risk factors. Cancer was not mentioned as a mortality factor in that cohort [3]. In addition, that investigation did not include osteoporosis, which is generally considered an important factor in morbidity and mortality in older adults [4, 5]. One report found a slightly higher mortality risk for osteoporosis than the expected general mortality risk. However, that study did not compare the risk of mortality from osteoporosis with other risk factors with respect to general health status [6]. Other studies have reported slightly higher (between 1.2 and 1.5) relative risks of mortality associated with an osteoporosis diagnosis [7–9], with the exception of one study from Japan that actually showed a higher mortality risk [10].

Studies investigating osteoporosis typically focus on fractures and the subsequent diagnosis of osteoporosis. Data on clinically asymptomatic individuals diagnosed with osteoporosis are scarce, although some studies are cited above.

Today, osteoporosis is known to be a disease of the elderly. However, the epidemiological frequency is underestimated, and its prevalence in some places, such as in Switzerland, is unknown. Another example of this underestimation is the treatment gap. In Switzerland, estimates suggest that between 36 and 58% of diagnosed cases of osteoporosis are not treated as recommended by the guidelines [11]. Epidemiological data from Sweden estimate that osteoporosis affects approximately 7.8% of men and 27.9% of women among citizens aged 70–74 years [12]. In Europe, the prevalence of osteoporosis is estimated to be between 5.9 and 7.2% in men and 19.1 and 23.5% in women [12].

One of the aims of the present prospective cohort study was to evaluate general health-impairing conditions in subjectively healthy retired people in Switzerland. This study also aimed to explore under-evaluated potential risk factors for mortality in this age group and to compare the magnitudes of their effects.

## Methods

### Aim, design and study setting

The present study involved a population-based prospective cohort. The study was conducted within the framework of the SENIORLAB [13] study (DOI 10.1186/ISRCTN 53778569). The primary aim of that study was to establish reference intervals for several laboratory parameters in an elderly cohort (www.seniorlabor.ch).

### Characteristics of the participants

**Fig. 1:**
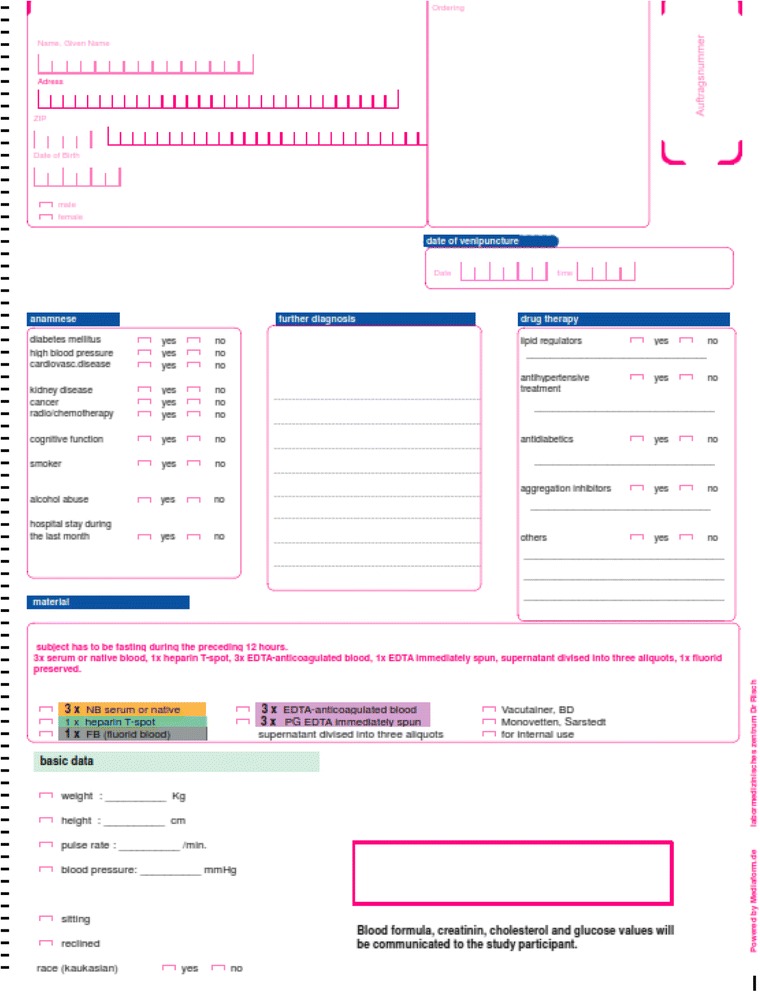
Study questionnaire as it was presented to study participants

The study consecutively enrolled subjectively healthy elderly volunteers from February 2009 to July 2012 and has been described in detail elsewhere [14, 15]. Potentially eligible participants were contacted via newspaper advertisements, various associations with high proportions of healthy elderly members (e.g., alpine and sports clubs), and personal contacts of the study collaborators. The inclusion criteria were as follows: at least 60 years of age, residence in Switzerland, and subjects describing themselves as healthy. Prior to study entry, the participants completed a questionnaire (Fig. 1). Subjectively healthy elderly were defined as seniors or pensioners who lived at home and could independently manage their daily lives without clinical signs of disease.

The present study did not apply any additional exclusion criteria to those used previously. We collected the subjects’ personal histories, anthropometric measurements (body weight, height, and body mass index [BMI]), and a fasting venous blood sample that was collected into S-Monovette tubes (Sarstedt, Sevelen, Switzerland). Consistent with standard regional habits in Switzerland, the food intake of the participants consisted of an approximate daily energy consumption per person of 2661 kcal (11,135 kJ), including 14% protein, 51% carbohydrate, and 35% fat. None of the participants was alcohol dependent. The participants were informed of selected results from their laboratory tests that were relevant for healthy seniors (i.e., their glucose, haemoglobin A1c (HbA1c), and creatinine levels). The risk of participation was considered very low with respect to the risk of phlebotomy of a peripheral vein (usually in the cubital region), which under Swiss ethics is classified as category A. The exclusion criteria were candidates who had overt type 2 diabetes mellitus (T2DM) or missing fasting plasma glucose (FPG) or HbA1c values.

Follow-up at the intercurrent end points of the study was conducted by directly contacting the study participants or their relatives or caregivers. Collection of death rates was conducted on behalf of the public authorities. The follow up visits ended in December 2014. The details of the SENIORLAB study have recently been published [16]. Importantly, co-morbidities obtained at baseline were included in the analysis.

### Data collection

At the baseline visit, subject’s histories and vital data such as height, weight and BMI were taken. The questionnaire given to the participants is presented in Fig. 1. In Addition, systolic and diastolic blood pressure was recorded in a sitting position after a 10-min rest. Venous blood was drawn into S-Monovette tubes after overnight fasting. Blood samples were processed (centrifuged, aliquoted, and analysed or frozen at − 80 °C) immediately to enable standardized preanalytics. A follow-up interview collected information on subjective well-being and survival. A negative response to the question of whether the patient was feeling healthy was defined to have unhealthy conditions, which was related to morbidity.

For the follow-up interview, the study subjects were contacted by mail and telephone. In cases with no response, official communal authorities, relatives, or neighbours were contacted. All diagnoses were obtained using good medical practices in doctor’s offices or by medical hospital staff. Where possible, osteoporosis was diagnosed using osteodensitometry.

Laboratory parameters were measured using various analytic platforms. We employed commercially available materials to guarantee quality control. The highly sensitive C-reactive protein, retinol-binding protein, α1-acid glycoprotein, and haptoglobin levels were determined using the Siemens ProSpec (Siemens, Zurich, Switzerland). The HbA1c level was measured using high-performance liquid chromatography (Bio-Rad D-10; Pratteln, Switzerland). Total cholesterol, low-density lipoprotein cholesterol, high-density lipoprotein cholesterol, blood urea nitrogen, uric acid, lipase, pancreatic amylase, prealbumin, and transferrin were determined on the Cobas Integra 800 (Roche Diagnostics Rotkreuz Switzerland). Measurements of brain natriuretic peptide and thyroid-stimulating hormone were obtained using the Architect i4000 instrument (Abbott, Baar, Switzerland). Parathyroid hormone and β2-microglobulin were assayed using the Immulite 2000 analyser (Diagnostics Products Corporation, Bühlmann Laboratories, Allschwil, Switzerland).

The isotope dilution mass spectrometry–standardized serum creatinine concentration was determined using a modified Jaffe method on the Cobas Integra 800 instrument. The inter-day coefficients of variation for creatinine were 4.27% at 42 μmol/L and 1.96% at 556 μmol/L.

The datasets used and/or analysed during the current study are available from the corresponding author upon reasonable request.

### Ethics

This study was performed in accordance with the ethical guidelines of the 1957 Declaration of Helsinki, and informed consent was received from all participants. Ethical approval for the present study was obtained from the Cantonal Ethics Committee of Bern (KEK Bern, Study Nr 166/08), Bern, Switzerland.

### Statistical analysis

The data analyses were done using the statistical software package Stata, version 6.0 for Windows (Stata Corp., 2000, College Station, TX, USA). Values are reported as the means ± standard errors of the mean. Differences were considered significant at *P* < 0.05.

**Fig. 2:**
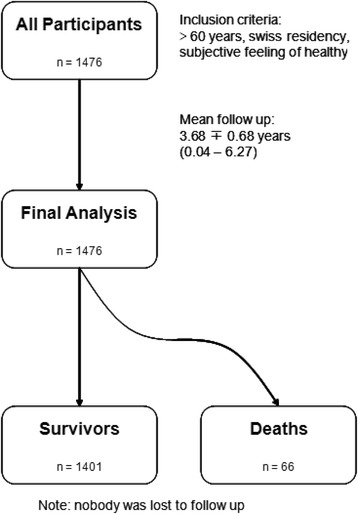
Presentation of study flow chart

The mean follow-up time for the entire cohort was 3.68 years. A flow chart of the study is shown in Fig. 2.

We analysed possible predictors, such as smoking, sex, cancer, cerebrovascular disease, heart disease, T2DM, and osteoporosis, using Cox regression analysis with and without covariates (Cox proportional hazards model), the Chi-square test, and Fisher’s exact test. Multivariate and Cox regression analyses with covariates were performed to assess the association of different possible risk factors detected by bivariate analysis and were incorporated into the final statistical model. Confounding variables were included if they changed the risk estimates by > 10%.

The Kaplan-Meier method was used for the survival analysis.

## Results

### Subject characteristics

The characteristics of the cohort are shown in Tables 1 and 2. In total, 1467 subjects were included in the cohort. The mean follow-up time was 3.68 years (95% confidence interval [CI], 3.64–3.71), and the median follow-up time was 3.83 years. The tables for women and men are provided separately. The ages of the participants ranged from 60 to 99 years. The ratio of men to women in the population was 1:1.16. The mean BMI was 25.5 kg/m^2^. The prevalence of osteoporosis in our cohort was 0.4% in men and 6.4% in women. Significant differences were found for weight, BMI, smoking status, type 2 diabetes, hypertension and osteoporosis. No significant differences were observed for age, cerebrovascular disease, cancer or death.

**Table 1:** Demographic characteristics of the SENIORLAB study: Men

Variable	Subjects	Mean, (%)	STD	Min; Max
age [years]	680	71.7	± 7.6	60;96
weight [kg]	679	79.5	± 12.0	50;176
BMI [kg/m^2^]	678	26.1	± 3.6	18.6;56.8
Follow-up [years]	680	3.60	± 0.77	0.04;6.27
Smoker	63	9.3		
Alcohol abuse	6	0.9		
Typ 2 diabetes mellitus	52	7.6		
Hypertension	285	41.9		
CVD	115	16.9		
Osteoporosis	3	0.4		
Cancer	48	7.1		
Deaths	37	5.4

**Table 2:** Demographic characteristics of the SENIORLAB study: Women

Variable	Subjects	Mean, (%)	STD	Min; Max
age [years]	787	72.4	± 8.1	60;99
weight [kg]	787	65.7	± 11.1	36;121
BMI [kg/m^2^]	785	24.9	± 4.0	14.4;42.9
Follow-up [years]	787	3.74	± 0.58	0.06;5.05
Smoker	37	4.7		
Alcohol abuse	0	–		
Typ 2 diabetes mellitus	18	2.3		
Hypertension	277	35.2		
CVD	109	13.9		
Osteoporosis	50	6.4		
Cancer	58	7.4		
Deaths	29	3.7		

Study population: number of patients, mean values (mean), standard deviation (STD), minimum (Min), maximum (Max), the difference will be the range, cerebrovascular disease (CVD)

There are statistical differences between men and women in weight, BMI, Follow-up, Smokerstatus, Typ 2 diabetes, hypertension, osteoporosis

No differences were detected in age, cerebrovascular disease, cancer and deaths

### Bivariate analysis

As of 2014, diabetic pensioners had a higher mortality risk than non-diabetics (hazard ratio [HR], 2.79; 95% CI, 1.38–5.66; *P* = 0.001). Participants previously diagnosed with osteoporosis had a higher mortality risk (HR, 3.01; 95% CI, 1.30–6.98; *P* = 0.01). A previous cerebrovascular incident resulted in a higher mortality risk (HR, 2.48; 95% CI, 1.45–4.25; *P* = 0.01). The bivariate calculated hazard ratio of mortality was 1.96 (95% CI, 1.21–3.19, *P* = 0.01) for hypertensive participants compared with non-hypertensive subjects. Active smokers did not have a significantly higher mortality rate than non-smokers. Participants who reported being diagnosed with cancer during the 6-year period before inclusion in the cohort did not have a higher mortality risk than those without a cancer diagnosis. For cancer, the follow-up time was 3.58 years (min. 0.26 years; max. 4.55 years). We obtained the same finding with respect to heart disease. In fact, we observed no higher mortality risk for any of the above conditions (Table 3).

**Table 3:** Primary risk factors and assignment as cause of death (*n* = 1467)

Risk Factor	Hazard Ratio	*p*	95% CI
Smoking	1.06	0.90	0.43–2.65
Hypertension	1.96	0.01	1.21–3.19
Cancer	1.59	0.25	0.73–3.49
Cerebrovasc. Disease	2.48	0.01	1.45–4.25
Heart Disease	2.04	0.13	0.82–5.08
Type 2 diabetes	2.79	0.01	1.38–5.66
Osteoporosis	3.01	0.01	1.30–6.98

Binary variables (smoking, hypertension, cancer, cerebrovascular disease, type 2 diabetes mellitus, osteoporosis) are given as hazard ratio’s. 95% CI: 95% Confidence Interval

### Multivariate analysis

The results of the multivariate analysis using the Cox proportional hazard model are shown in Table 4. Diabetes was a stable risk factor with a HR of 2.17. A slight confounding effect was evident in the bivariate analysis. Our model demonstrated that osteoporosis was a risk factor for mortality, although it was underestimated in the bivariate analysis (HR, 4.46). Other important factors that influenced mortality were hypertension (OR, 1.81), age (HR, 1.10), and female sex (HR, 0.48).

**Table 4:** Cox proportional hazards model for mortality

Risk Factor	Hazard Ratio	*p*	95% CI
Hypertension	1.81	0.02	1.09–3.03
Type 2 diabetes	2.17	0.04	1.04–4.52
Osteoporosis	4.46	0.01	1.82–10.91
Age	1.10	0.01	1.04–1.17
Age*Age	1.003	0.04	1.001–1.006
Female Gender	0.48	0.01	0.28–0.81

Age*Age: Age has a square function in the model. 95% CI: 95% Confidence Interval

Type 2 diabetes, hypertension, osteoporosis, age, and female sex were important predictors in the model with death as the outcome variable

### Survival analysis

**Fig. 3:**
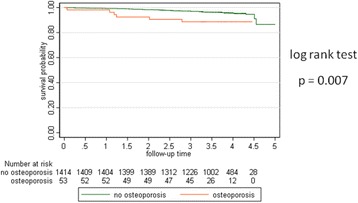
Kaplan Meier curves show a statistical significant difference. Mean Follow up time was 3.68 years (95% CI 3.64–3.71 years)

Survival analysis obtained by the Kaplan Meier Method showed a statistical reduced life expectancy in patients having osteoporosis (see Fig. 3).

### Influence of a low BMI and vitamin D3 serum level on osteoporosis

**Fig. 4:**
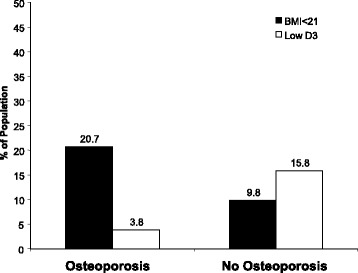
Low body mass index (BMI) was defined as < 21 kg/m^2^. In participants diagnosed with osteoporosis, 20.7% had a low BMI compared with 9.8% in the non-osteoporotic sub-cohort (*P* < 0.05, Wilcoxon); 25-hydroxyvitamin D3 serum levels < 13 ng/ml were observed in 3.8% of subjects in the osteoporotic sub-cohort compared with 15.8% in those with no diagnosis of osteoporosis

Of the participants diagnosed with osteoporosis, 20.7% had a BMI lower than 21 kg/m^2^ compared with 9.8% in the non-osteoporotic sub-cohort *(P <* 0.01, Chi^2^). A 25-hydroxvitamin D3 serum level < 13 ng/mL was observed in 3.8% of the subjects in the osteoporotic sub-cohort compared with 15.8% in those without an osteoporosis diagnosis *(P <* 0.02, Chi^2^). (see Fig. 4).

## Discussion

This report describes mortality and associated risk factors in an older Swiss population. Beyond the effects of these risk factors on mortality, a central concern is the time lag between their identification and the occurrence of death. As of March 2017, 1401 study participants survived, and 66 had died. Fortunately, that number is relatively low; however, these data allow us to employ health risk management techniques when assessing the potential causes of death for target prevention.

In addition to T2DM, osteoporosis was a strong predictor of mortality in our senior cohort. This finding was evident in the bivariate analysis (Table 3) and our survival analysis. In our multivariate model using Cox regression models with covariates (Table 4), osteoporosis had a higher relative risk of mortality than type 2 diabetes. The relative risk of osteoporosis seems to be high. Based on the absolute numbers, we assumed that osteoporosis in our cohort was responsible for approximately 6% of deaths; hypertension was responsible for 26% of deaths and diabetes was responsible for 9% of all fatalities. Although comparisons with other mortality risk factors in a cohort of healthy senior citizens are lacking, osteoporosis has been identified in several studies as an important risk factor for all-cause mortality. Our data confirmed the results of a Danish cohort study, which found a shorter life expectancy among osteoporosis patients. One conclusion of the present study was significant comorbidity in such patients with a higher mortality risk [14]. Additional evidence for osteoporosis as an important all-cause mortality risk factor comes from studies in the Netherlands [15], United Kingdom [17], and Austria [18]; however, those studies were conducted retrospectively. In addition, the relatively low prevalence of osteoporosis in our cohort compared to that in the general Swedish population in the same age group (osteoporosis prevalence rates of 7.8% in men and 27.9% in women) would predict a much higher death rate and most likely a higher public health relevance than was found in our study. Based on our results, we propose that osteoporosis is relevantly underreported in the study population, which may lead to an underestimation of its public health relevance compared to diseases such as hypertension.

Why might osteoporosis lead to a higher mortality rate? The mechanism is complex and multifactorial, and the precise pathways are not known. As shown by Sakem and coworkers, a low vitamin D level is involved in an altered immune response [13]. However, we found no direct association between vitamin D and all-cause mortality and osteoporosis; specifically, 3.8% of osteoporosis patients had high vitamin D levels, whereas 15.8% of patients without osteoporosis had a vitamin D level of less than 13 ng/mL (Fig. 4). In contrast to the results for 25-hydroxyvitamin D3 levels, 20.7% of our participants with low BMIs (defined as less than 21 kg/m^2^) were in the osteoporotic group compared with only 9.8% in the non-osteoporotic population. This result indicates that an impaired nutritional state could be reflected by osteoporosis. This finding supports the results of a US study by Thomas-John et al., which reported that osteoporosis was associated with weight loss and low consumption of dairy products, suggesting that malnutrition might be an important cofactor [19]. In addition, an Australian longitudinal study observed a clinically relevant reduction in mortality among older seniors diagnosed with osteoporosis without fractures undergoing bisphosphonate treatment [20].

The most prevalent contemporary causes of death are cardiovascular and cerebrovascular disorders and cancer. Recent decades have seen rapid advances in diagnoses and surgical and pharmaceutical therapies for these conditions. Comparative studies of mortality in Europe have shown that the all-cause mortality per 1000 people aged 70–74 years is 25.1 for women and 47.1 for men [21]. The all-cause mortality rates in Alaska and Florida were estimated to be 43.5 and 44.2, respectively. In our cohort (mean age, 72.1 years; 95% CI, 71.7–72.5), the overall mortality was 12.2 per 1000. In the present study of an apparently healthy Swiss population of pensioners, we found that T2DM, osteoporosis, and hypertension were important risk factors for mortality.

Unsurprisingly, hypertension and T2DM are leading causes of mortality, as shown by the Cardiovascular Health Study [3]. The prevalence of T2DM is greatest in people over 65 years of age, and the highest incidence also occurs in that age group [22–24]. Some authors believe that the burden of diabetes in the older generation will increase further due to demographic changes, the rising incidence of T2DM in all groups [22], and the longer survival times of people diagnosed with diabetes at a younger age [25]. However, older people are under-represented in clinical trials; thus, they are often treated according to guidelines based on expert opinions and extrapolation of results obtained from trials on younger people [26]. People diagnosed with T2DM at an older age account for approximately half of all new cases. Consequently, diabetes and mortality data in older populations have produced heterogenic results and have led to a mortality risk estimation with an OR of 3.0 [27] but only a slight, non-significant risk elevation [28]. One systematic review demonstrated a pooled OR of 1.4 for mortality and T2DM in an elderly population over 65 years of age [29]. Our data from this population-based prospective cohort indicate that T2DM is a major risk factor for death, even for the older generation. Diabetes appears to be a leading cause of arteriosclerosis and death, even with the relatively short follow-up period of 3.7 years used by this study.

In addition to diabetes, hypertension is an independent predictor of mortality in elderly populations. This result is not surprising and has been convincingly demonstrated by multiple studies [30–32]. The magnitude of its effect is associated with the degree of hypertension [26]. Weidung et al. showed that mortality due to hypertension increased in older adults who were active and robust [27]. We assumed that the individuals in our cohort were robust, since being subjectively healthy was a requirement for inclusion in the study. Huynh et al. found that hypertension was less of a risk factor for all-cause mortality than T2DM. In their models obtained from a cohort of older adults, T2DM was responsible for 50% of cardiovascular mortality, although it may have overlapped with the effect of hypertension [33]. A similar magnitude of mortality risk was reported in the multivariate models of Fried et al. in the Cardiovascular Health Study [3].

We did not find that a history of cancer was a predictor of death. That result was expected, since the observational time was too short to demonstrate an effect of cancer on all-cause mortality. In this context, comorbidities other than cancer play more important roles in all-cause mortality in older patients diagnosed with cancer, at least in cases with a relatively short surveillance period [34], such as the present interim report.

One strength of the present study is its prospective design and follow-up of 3.7 years in subjectively healthy seniors. A weakness is the lack of osteodensitometry measurements to define the disease. Additionally, our data are based on self-reporting and may have underestimated conditions. On the other hand, in the absence of other representative samples, this study provides the best available estimates, as stated elsewhere [35]. Thus, our results may have overestimated the association between osteoporosis and mortality in this older population as a result of recall bias. Osteoporosis and malnutrition may be easily treated, but they have large impacts on public health. We believe that osteoporosis in the elderly may be under-diagnosed as a result of reduced physician awareness of the condition. Moreover, our population showed a smaller prevalence of osteoporosis than the reported prevalence in Sweden (women, CH: 6.4%; women SWE: 27.9%). This finding suggests underreporting in our sample. Diagnosing osteoporosis should be undertaken more proactively in older people with subjectively good health.

## Conclusions

We demonstrated that hypertension and T2DM followed by osteoporosis were major factors of all-cause mortality in subjectively healthy seniors. Osteoporosis is probably underestimated due to underreporting and underestimation by clinicians. Osteoporosis should be more actively diagnosed in healthy older people before they experience osteoporosis-associated incidents.

## Abbreviations

BMI: Body mass index
CI: Confidence interval
Fig.: Figure
FPG: Fasting plasma glucose
HbA1c: Haemoglobin A1c
OR: Odds ratio
T2DM: Type 2 diabetes mellitus
